# Development of a Mobile Assessment Tool for Understanding Social Comparison Processes Among Individuals With Schizophrenia: Two-Phase Survey Study

**DOI:** 10.2196/36541

**Published:** 2022-05-02

**Authors:** Danielle Arigo, John Torous

**Affiliations:** 1 Department of Psychology Rowan University Glassboro, NJ United States; 2 Department of Family Medicine Rowan School of Osteopathic Medicine Stratford, NJ United States; 3 Beth Israel Deaconess Medical Center Harvard Medical School Boston, MA United States

**Keywords:** schizophrenia, social comparison, mobile assessment, smartphone app, variability

## Abstract

**Background:**

Digital tools may help to address social deficits in schizophrenia, particularly those that engage social comparison processes (ie, evaluating oneself relative to others). Yet, little is known about social comparison processes in schizophrenia or how best to capture between- versus within-person variability, which is critical to engaging comparisons in digital interventions.

**Objective:**

The goals of this pilot study were to (1) better understand affective responses to social comparisons among individuals with schizophrenia, relative to healthy controls, using a validated global self-report measure; and (2) test a new brief, mobile assessment of affective responses to social comparison among individuals with schizophrenia, relative to the full measure. This study was conducted in 2 phases.

**Methods:**

We first compared self-reported affective responses to social comparisons between individuals with schizophrenia (n=39) and healthy controls (n=38) using a traditional self-report measure, at 2 time points. We examined the temporal stability in responses and differences between groups. We then evaluated the performance of brief, mobile assessment of comparison responses among individuals with schizophrenia, completed over 12 weeks (n=31).

**Results:**

Individuals with schizophrenia showed greater variability in affective responses to social comparison than controls on traditional measures and completed an average of 7.46 mobile assessments over 12 weeks. Mobile assessments captured within-person variability in affective responses in the natural environment (intraclass correlation coefficients of 0.40-0.60). Average scores for mobile assessments were positively correlated with responses to traditional measures.

**Conclusions:**

Affective responses to social comparison vary both between and within individuals with schizophrenia and capturing this variability via smartphone surveys shows some evidence of feasibility. As affective variability is a potential indicator of poor outcomes among individuals with mental health conditions, in the future, a brief, mobile assessment of affective responses to social comparisons may be useful for screening among individuals with schizophrenia. Further research on this process is needed to identify when specific comparison messaging may be most effective in digital interventions and could suggest new therapeutic targets for illnesses such as schizophrenia.

## Introduction

Schizophrenia currently affects approximately 1% of the US population [1]. Available pharmacological treatments can address positive symptoms (eg, hallucinations, delusions), but they are not as effective for negative symptoms (eg, amotivation, social deficits) [2], which are associated with greater disability and impairment [3]. While specialized therapies exist to help treat the social deficits in schizophrenia, access to them remains limited [4], and understanding ways to encourage social engagement remains a core priority for research and clinical care.

Across health care, digital technologies have the potential to increase access to and improve quality of care. Digital treatments for mental health conditions, such as those delivered via smartphone apps, are popular and over 10,000 already exist [5]. However, sustained engagement with app-based treatments is low: after 2 weeks, 96% of those who download a mental health app have stopped engaging with it [6]. Given the relapse, remitting, or chronic pattern of mental illnesses such as schizophrenia, sustained engagement is critical for digital treatments to have maximal impact. Strategies to boost engagement with these treatments include use of social networks to promote social support [7]. Evidence shows that social networks are currently the most effective means to drive sustained engagement with mental health apps, and that apps that offer social support have higher rates of engagement than those that do not [6].

In addition to processes such as social support, the efficacy of social networks to drive engagement (and consequent behavior change) rests in part on *social comparison* processes. Comparing one’s opinions, skills, or behaviors to those of relevant others allows people to evaluate themselves, which reduces uncertainty in these domains [8]. Theory and evidence indicate that people make *upward comparisons* (comparing themselves to someone better off) or *downward comparisons* (comparing themselves to someone worse off), and that these comparisons can have a range of consequences for both short-term affect and longer-term behavior [9]. The effects of comparisons depend on a variety of person-level and contextual factors, including perceived similarity to the comparison target [10].

Specifically, the Identification/Contrast Model [11], which has been applied often in chronic illness populations, suggests that focusing on similarities between the self and an upward comparison target (*upward identification*) leads to positive affective responses, such as increased confidence in one’s ability to achieve the target’s status and motivation to engage in related behaviors. Focusing on differences between the self and an upward target (*upward contrast*) has the opposite effect, as it highlights the comparer’s inferiority and may suggest that a similar status is not achievable. Conversely, focusing on similarities with a downward target (*downward identification*) leads to negative affective responses, as this confirms that the comparer’s situation is or will become grave. Focusing on differences between the self and a downward target (*downward contrast*) can alleviate anxiety and boost positive affect, as the comparer is already doing better than someone else.

In addition, there is ample evidence that people with mental health conditions (eg, major depression, anxiety disorders) may use and respond to comparisons differently than people without these conditions [12,13], though the extent to which identification and contrast processes contribute to these differences is unknown. Specifically, within-person *variability* in affective response associated with identification versus contrast may help to explain these differences. Greater (vs. lesser) affect variability is associated with poorer mental health outcomes, such as lower self-esteem, worse depressive symptoms, and more neuroticism [14], as well as more frequent alcohol use [15]. A better understanding of variability in affective responses to social comparison in schizophrenia would be useful for treatment, given the disease-specific needs to improve social outcomes and general needs to improve engagement with digital treatments (eg, mobile apps) that afford the potential of scalable and accessible care. Social comparison offers a theoretical basis with real-world applicability to drive engagement with digital treatments in this population.

Although both upward and downward comparisons are common in illnesses such as cancer [10], prior research suggests that those with schizophrenia predominantly use downward comparisons [16], and that downward comparisons may propagate delusional states [17]. However, this research is limited in scope, and to date, the topic has received little attention. Further, despite the frequency with which social comparison is cited as a feature of digital health apps [18], little is known about *how* social comparison drives engagement and outcomes with apps, especially apps designed for chronic mental illness. Understanding the impact of social comparison in this context is critical, as negative uses of social comparison (eg, upward contrast or downward identification) could reduce app engagement and motivation for healthy behavior, while positive use (eg, upward identification or downward contrast) could drive sustained engagement and healthy behaviors. At present, however, it is not clear how best to assess patients’ identification and contrast processes in the context of a digital environment.

Effective assessments should be ecologically valid and respond to known contextual influences on social comparison processes [19]—specifically, that they will capture variability in responses for the same person over time, as well as differences between people that are more stable over time [20]. This distinction is particularly necessary for examining affective variability and has been identified as critical to advancing clinical science in schizophrenia [21,22]. Ideally, these assessments also would be brief and conducive to integration with other app features, to allow these features to adapt to immediate or longer-term shifts in response to social comparisons. As no such assessment tool exists, the aims of this pilot study were to differentiate between- versus within-person variability in responses to a global social comparison measure among individuals with schizophrenia and healthy controls, and to examine the performance of a brief, mobile version of this measure among individuals with schizophrenia. The research questions and exploratory hypotheses that guided this study were:

1. How do self-reported responses to social comparisons among individuals with schizophrenia compare with those of healthy controls? We expected to observe stronger negative or weaker positive responses to comparisons among those with schizophrenia.
2. A. How much within-person variability is there in self-reported responses to social comparisons? We expected to observe meaningful within-person variability in affective responses to social comparisons.
  B. Does variability differ between individuals with schizophrenia and healthy controls? We expected to observe greater within-person variability in affective responses to social comparisons among individuals with schizophrenia.
3. Among individuals with schizophrenia, does a brief mobile assessment of self-reported responses to social comparisons show convergent validity with the full scale? We expected to observe moderate to strong correlations between scores on the full and mobile versions of this measure.

## Methods

### Participants and Procedure

Procedures were approved by the institutional review board at the supporting institution and all participants provided written informed consent. Adults with schizophrenia were recruited from outpatient clinics in a large city in the northeastern United States region, where diagnosis was verified through clinical records. Control participants were recruited via online social media postings targeting college students in the same city. Control participants were assessed in person and were eligible if they did not screen positive for mental illness based on the Mini International Neuropsychiatric Interview [23]. Smartphone ownership and ability to run the study app on that smartphone, age 18 or older, and ability to participate in informed consent processes were the inclusion criteria in both samples.

Participants were 39 patients with schizophrenia (20/39, 51%, men; mean age 37.45 [SD 14.86] years) and 38 healthy controls (17/38, 45%, men; mean age 30.50 [SD 16.65] years; Table 1). As part of a larger clinical battery, all participants completed the full Identification-Contrast Scale (described below) in the clinic, both at the start of the study and at the second visit 3 months later. A total of 59 participants returned for the 3-month follow-up (n=31 patients, n=28 healthy controls); rates of attrition did not differ between samples (χ^2^_1_=0.05; *P*=.82). All participants were compensated for in-person assessments at US $20 per visit.

During 12 weeks of mobile assessment that occurred between clinic visits, the mobile version of the Identification-Contrast Scale developed for this study (also described below) appeared twice per week, among patients with schizophrenia only. A total of 24 patients completed mobile assessments during the 12-week window. Participants were oriented to the questions in person. When using the app between sessions, participants were free to ignore any mobile surveys and were not compensated on the basis of completion.

**Table 1:** Demographic information for individuals with schizophrenia and healthy controls.

Demographic		Individuals with schizophrenia (n=39)	Healthy controls (n=38)^a^
Age, mean (SD)		37.45 (14.86)	30.50 (16.65)
**Gender, n (%)**			
	Men	20 (51)	17 (45)
	Women	19 (49)	19 (50)
**Race, n (%)^b^**			
	American Indian or Alaskan native	4 (10)	0 (0)
	Asian American	1 (3)	25 (66)
	Black or African American	11 (28)	3 (8)
	Multiracial or other	1 (3)	2 (5)
	White	21 (54)	6 (16)
**Education, n (%)**			
	Four-year college graduate or higher	14 (36)	30 (79)
	Some college	11 (28)	3 (8)
	High-school graduate/General Educational Development	11 (28)	3 (8)
	Some high school	3 (8)	0 (0)

^a^Two participants did not provide complete demographic information.

^b^One participant did not specify their race.

### Full Measure (All Participants)

The Identification-Contrast Scale [24] is a 12-item measure of positive and negative responses to comparisons with upward and downward targets, allowing for inferences about identification and contrast with each directional target. The measure has subscales for each direction and type of response (upward identification, upward contrast, downward identification, and downward contrast), with 3 items per subscale. Items such as “When I see or think about others who are doing better than I am, I am pleased that things can get better” are rated on a scale of 1 (*not at all*) to 5 (*strongly*). Responses for the 3 items associated with each subscale are averaged to create subscale scores; higher scores on each subscale indicate stronger perceptions of identification or contrast with the relevant target (upward vs. downward). This measure has shown strong psychometric properties among individuals with chronic conditions such as cancer [25] and traumatic brain injury [26]. In this study, internal consistency estimates (Cronbach α) across all participants at baseline were high for all subscales: .84 for upward identification, .78 for upward contrast, .83 for downward identification, and .85 for downward contrast.

### Mobile Assessment (Individuals With Schizophrenia Only)

The mobile version of the Identification-Contrast Scale was designed to maximize the power of the full scale while limiting the number of items to be completed in the natural environment. To achieve this balance, the item on each scale with the highest factor loadings was selected for delivery via mobile app [24]. These were *When I see or think about others who are doing better than I am, I realize that it’s possible to improve* (upward identification); *When I see or think about others who are doing better than I am, I feel frustrated about my own situation* (upward contrast); *When I see others who are doing worse than I am, I feel fear that my future will be similar to them* (downward identification); and *When I see others who are doing worse than I am, I feel relieved about my own situation* (downward contrast). We retained the exact wording of these items to maintain their validity. During orientation to the measures, however, participants were instructed to respond to these items with their recent (rather than global or aggregated) experiences; specifically, they were asked to focus on their experiences since the last assessment.

### Data Analysis

Descriptive statistics for each subscale of the full Identification/Contrast measure included means and SDs for each group (individuals with schizophrenia vs. healthy controls) at each time point. To address the first research question, independent *t* tests with associated Cohen d effect sizes were used to compare scores between groups at each time point. With respect to the second research question, 2-level multilevel models with restricted maximum likelihood estimation were used to account for assessment points (level 1) nested within individuals (level 2). Intraclass correlation coefficients (ICCs) were calculated from empty models to determine the proportions of variance attributable to stable, between-person differences and within-person variation (plus error; research question 2A), and differences between groups were tested with model comparisons (χ^2^; research question 2B).

The third research question was addressed in 2 ways. First, descriptive information was examined to determine how often individuals with schizophrenia completed mobile assessments of social comparison responses and how much variability in their responses was between- versus within-person. Second, bivariate correlations (*r*) were calculated between full-scale scores and the average of each participant’s brief mobile assessments. Given the small sample size for this preliminary study, particularly for individuals with schizophrenia who completed mobile assessments (n=24), the criterion for statistical significance was set at *P*<.10, and effect size estimates were emphasized for interpretation of findings.

### Ethical Approval

The Institutional review board at Beth Israel Deaconess Medical Center has approved this study (institutional review board protocol number: 2017P000359).

## Results

### Identification and Contrast Among Individuals With Schizophrenia Versus Healthy Controls

Descriptive statistics for each group by time point are presented in Table 2. At time 1, individuals with schizophrenia reported stronger tendencies toward upward contrast (t_76_=2.82, *d*=0.63) and downward identification (t_76_=3.10, *d*=0.69) than healthy controls (*P*s<.01), and both differences were associated with medium effect sizes. At time 2, the group difference for downward identification persisted (t_56_=2.66, *d*=0.71; *P*=.01), and a group difference for downward contrast emerged (ie, individuals with schizophrenia reported weaker tendencies; t_56_=–2.35, *d*=0.63; *P*=.02). However, the group difference for upward contrast disappeared at time 2 (t_56_=1.54; *P*=.13). Groups did not differ with respect to upward identification at either time point (*P*s>.57). Thus, for 3 of 4 subscales, individuals with schizophrenia reported stronger tendencies toward negative-outcome comparisons, and weaker tendencies toward positive-outcome comparisons than did healthy controls.

**Table 2:** Descriptive statistics for traditional self-report measures and differences between individuals with schizophrenia and healthy controls.

Response to comparison			Individuals with schizophrenia, mean (SD)		Healthy controls, mean (SD)		Differences between samples
**Time 1^a^**							
	Upward identification	4.06 (1.08)		4.19 (0.99)		t_76_=–0.57, *d*=0.13	
	Upward contrast	2.54 (1.35)		1.81 (0.92)		t_76_=2.82^b^, *d*=0.63	
	Downward identification	2.06 (1.26)		1.37 (0.55)		t_76_=3.10^b^, *d*=0.69	
	Downward contrast	3.36 (1.26)		3.78 (0.97)		t_76_=1.69, *d*=0.37	
**Time 2^c^**							
	Upward identification	3.92 (1.11)		4.01 (0.86)		t_56_=0.33, *d*=0.09	
	Upward contrast	2.57 (1.29)		2.07 (1.18)		t_56_=1.54, *d*=0.40	
	Downward identification	2.22 (1.26)		1.49 (0.71)		t_56_=2.66^d^, *d*=0.71	
	Downward contrast	2.88 (1.42)		3.62 (0.91)		t_56_=–2.35^d^, *d*=0.63	

^a^n=39 and 38 for columns 2 and 3, respectively.

^b^*P*<.01.

^c^n=31 and 28 for columns 2 and 3, respectively.

^d^*P*<.05.

### Variability in Identification and Contrast

Across time points and participant groups, ICCs for upward and downward identification were 0.40 and 0.41, respectively, indicating that approximately 40% of variability in these tendencies was due to stable, between-person differences, whereas 60% was due to within-person variation (and error). Stability estimates for upward and downward contrast were slightly higher (ICCs 0.60 and 0.57, respectively), though within-person variation components for all 4 scales were statistically significant (*P*s<.01). Moreover, individuals with schizophrenia showed greater variability in responses to social comparison than healthy controls on 3 of 4 subscales (upward contrast: χ^2^_1_=8.20; downward identification: χ^2^_1_=25.70; downward contrast: χ^2^_1_=8.70; *P*s<.03). The exception was for upward identification (χ^2^_1_=1.50; *P*=.50), where variability did not differ between groups.

### Brief Mobile Assessment of Identification and Contrast

Among individuals with schizophrenia, there was considerable between-person variability in the number of mobile assessments of social comparison responses completed during the 12-week assessment window. These individuals completed assessments between 1 and 28 times, with an average of 7.46 times per person (SD 6.47). ICCs showed that 40%-60% of variability in response to each item was attributable to stable, between-person differences (Table 3), with the remaining 40%-60% capturing within-person variation across assessments and error. Between-person, average scores for mobile assessments of social comparison responses were positively correlated with responses to the same items when they were completed as part of the full measures (ie, at times 1 and 2). The strength of these associations ranged from *r*=0.17 to 0.72 (*P*s<.10). Mobile assessment of downward contrast showed the most consistent positive associations, with *r*=0.55 at time 1 and *r*=0.66 at time 2 (*P*s<.02). Moreover, scores on 1-item assessments were positively correlated with subscale scores on the full measures, with the strength of associations ranging from *r*=0.24 to 0.76 (Table 3).

**Table 3:** Variability estimates for mobile social comparison response measure and relations with traditional self-report measure among individuals with schizophrenia (n=24).

Response to comparison	Variability estimate (intraclass correlation coefficient)	Relation with time 1 score (*r*)	Relation with time 2 score (*r*)
Upward identification	0.40	0.38^a^	0.24
Upward contrast	0.60	0.53^b^	0.33
Downward identification	0.41	0.40^a^	0.76^c^
Downward contrast	0.57	0.50^b^	0.74^c^

^a^*P*<.10.

^b^*P*<.05.

^c^*P*<.01.

## Discussion

Individuals with schizophrenia experience meaningful deficits in social integration and perception that may be targeted with digital interventions, though patient engagement with these interventions is modest. The opportunity to make social comparisons may help to address these problems, though this concept has received little attention in schizophrenia. As an initial step, the results of this study provide necessary, if preliminary, insight into this process at multiple levels. The limited existing work on social comparisons among individuals with schizophrenia focused on the use of upward versus downward comparisons [16]. As both upward and downward comparisons can have positive and negative consequences [27], however, this study extended previous work by focusing on *responses* to upward and downward comparisons, rather than on their mere occurrence or frequency.

Specifically, this study captured reports of affective responses to upward and downward social comparisons (as indicators of identification and contrast processes), which are better longitudinal predictors of clinical outcomes among individuals with chronic medical conditions than the reported direction of comparisons [26]. Our findings show that patients with schizophrenia report experiencing negative affect from comparisons more often than healthy controls, and that this difference persists over 3 months. If these findings are confirmed in larger samples, clinical implications include (1) considering discussion of social comparisons in therapy sessions with patients as a potential trigger for their symptoms, and (2) providing guidance in digital interventions to minimize negative effects. Research implications include using comparisons to increase positive engagement with digital health interventions (eg, smartphone app notifications) and understanding whether social comparisons moderate negative symptoms in schizophrenia, depending on the environmental and social context.

Further, although most studies of social comparison focus on stable differences between people [9,10], the present findings underscore the dynamic nature of social comparison and suggest the value of repeated assessment for revealing how comparisons also vary over time and across contexts—particularly among individuals with chronic conditions such as schizophrenia. In addition to showing more frequent negative responses to comparisons than healthy controls, patients with schizophrenia showed greater *variability* in their negative and positive affective responses to comparisons over 3 months. Given that affect variability has been linked to poor mental health outcomes [14,15], it is possible that affect variability in response to comparisons in schizophrenia contributes to the maintenance of social deficits and related negative symptoms. This hypothesis requires further investigation.

Importantly, findings from this study also provide preliminary support for the feasibility of collecting real-time data on social comparison responses through digital tools such as apps, and suggest the potential for these data to inform the tailoring of digital interventions for schizophrenia. For example, although there were considerable between-person differences in the number of social comparison smartphone assessments completed (and considerable variability in item responses), smartphone assessments showed 3 important features. These assessments were voluntarily completed throughout the assessment period; they captured both between- and within-person variability in affective responses to comparisons; and responses to mobile items correlated with those completed with traditional self-reports from the original measure. Thus, a brief, smartphone-based assessment of social comparison responses appears to perform well for its intended purpose, and additional work is needed to confirm and extend these findings.

Overall, the observed variability in affective response to comparisons among patients with schizophrenia suggests that there are times when negative (and positive) affective responses are stronger than others. In future studies of this kind, smartphone-based assessment may enable modeling of moderators of social comparison response, such as comparison dimension (ie, what about the self is being compared), mode of comparison (ie, face-to-face vs. via social media), or motivation for comparison (ie, self-selected from a range of options for a particular purpose, or in response to exposure to a single target) [19]. Such an assessment also could be paired with passive data from smartphone sensors (eg, about sleep patterns, mobility, location) to help determine when a patient is likely to respond positively or negatively to a specific type of comparison, and thus, whether a comparison might have immediate utility. Together, this approach may enable more personalized models of social comparison that are tailored to the dynamic, real-time state of each patient, thus enabling more actionable decision points for use in just-in-time adaptive interventions [28]. Such tailoring is likely to promote engagement with digital interventions by more effectively responding to immediate needs, and thus, customizing a comparison opportunity that is most likely to be engaging or helpful to that person at the time it is deployed.

In the current era of socially connected digital health tools, where patients with schizophrenia engage at rates equal to the general population [29], there is a renewed need to understand how social comparison theory can assist in ensuring that technology-mediated social interactions are engaging and beneficial. In addition, the need to increase engagement with digital therapies is broader than the context of schizophrenia [28]. Although the results of this study offer insights into social comparison processes in schizophrenia, the methodology presented should be generalizable across many diverse use cases. Thus, the potential of social comparison processes to help drive engagement through more meaningful, relevant, and beneficial messaging that are responsive to local environmental, temporal, and social circumstance highlights the broad applicability of our novel methods.

Strengths of this study include its recruitment of both individuals with schizophrenia and healthy controls, both with equal proportions of men and women, and the use and comparison of both traditional self-report measures and brief versions modified for mobile assessment. Further, the emphasis of this study was on differentiating between- and within-person variability in a critical but understudied aspect of social comparison (ie, affective response), using appropriately sophisticated statistical methods.

As this was a formative pilot study, however, there were noteworthy limitations. Our sample sizes were modest, particularly at time 2, and participants were predominantly White or Asian American. We also did not have the opportunity to include a clinical control group. Given that participants had flexibility in their completion of mobile assessments, compliance with these assessments was inconsistent across participants. Modest compliance with mobile assessments is common among individuals with schizophrenia and other severe and persistent mental illnesses [30-33]. To ensure that missing data do not bias conclusions, a priority for future work will be to understand patterns of missingness and best practices for reducing it in these and similar populations. For example, participants were not compensated for completing assessments and did not have access to their survey data; adding these components may increase compliance with mobile assessments among individuals with schizophrenia.

In addition, despite reviewing instructions with participants at orientation to specify the time window they should use for reference when completing the mobile version of the Identification-Contrast Scale, it is possible that participants with schizophrenia responded with more global than contextually sensitive impressions of their affective responses. The considerable within-person variability observed in their responses suggests that the measure was sensitive to context, but future studies should consider adding more specific instructions to the mobile version of the measure.

Finally, given the complexity of social comparison and the emphasis on general affective responses in this study, assessments also did not capture all of the aspects of this process that may be relevant to its role in daily life. For example, measures used in this study did not assess individual instances of social comparison, and thus, did not capture the dimension or mode [34]. Nevertheless, as an initial step in this line of work, findings from this study provide critical evidence to inform future research focused on mobile assessment of social comparison and the tailoring of comparison opportunities to promote patient engagement with digital interventions.

## Abbreviations

ICC: intraclass correlation coefficient

## Data Availability

Data are available by request to the second author.
